# A novel small-molecule IAP antagonist, AZD5582, draws Mcl-1 down-regulation for induction of apoptosis through targeting of cIAP1 and XIAP in human pancreatic cancer

**DOI:** 10.18632/oncotarget.4822

**Published:** 2015-08-06

**Authors:** Jai-Hee Moon, Jae-Sik Shin, Seung-Woo Hong, Soo-A Jung, Ih-Yeon Hwang, Jeong Hee Kim, Eun Kyoung Choi, Seung-Hee Ha, Jin-Sun Kim, Kyoung-Mok Kim, Dae-Won Hong, Daejin Kim, Yeong Seok Kim, Jeong Eun Kim, Kyu-Pyo Kim, Yong Sang Hong, Eun Kyung Choi, Jung Shin Lee, Maureen Hattersley, Dong-Hoon Jin, Tae Won Kim

**Affiliations:** ^1^ Innovative Cancer Research, Asan Institute for Life Science, University of Ulsan College of Medicine, Asan Medical Center, Songpa-gu, Seoul, Republic of Korea; ^2^ Department of Oncology, University of Ulsan College of Medicine, Asan Medical Center, Songpa-gu, Seoul, Republic of Korea; ^3^ Department of Radiation Oncology, University of Ulsan College of Medicine, Asan Medical Center, Songpa-gu, Seoul, Republic of Korea; ^4^ Department of Convergence Medicine, University of Ulsan College of Medicine, Asan Medical Center, Songpa-gu, Seoul, Republic of Korea; ^5^ Department of Internal Medicine, University of Ulsan College of Medicine, Asan Medical Center, Songpa-gu, Seoul, Republic of Korea; ^6^ University of Ulsan College of Medicine, Seoul, Republic of Korea; ^7^ Department of Anatomy, Inje University College of Medicine, Busanjin-ku, Busan, Republic of Korea; ^8^ Oncology iMed, Innovative Medicines & Early Development, AstraZeneca R&D Boston, 35 Gatehouse Drive, Waltham, Massachusetts, USA

**Keywords:** IAP antagonist, Mcl-1, AZD5582, apoptosis, pancreatic cancer cells

## Abstract

Inhibitor of apoptosis proteins (IAPs) plays an important role in controlling cancer cell survival. IAPs have therefore attracted considerable attention as potential targets in anticancer therapy. In this study, we investigated the anti-tumor effect of AZD5582, a novel small-molecule IAP inhibitor, in human pancreatic cancer cells. Treating human pancreatic cancer cells with AZD5582 differentially induced apoptosis, dependent on the expression of p-Akt and p-XIAP. Moreover, the knockdown of endogenous Akt or XIAP via RNA interference in pancreatic cancer cells, which are resistant to AZD5582, resulted in increased sensitivity to AZD5582, whereas ectopically expressing Akt or XIAP led to resistance to AZD5582. Additionally, AZD5582 targeted cIAP1 to induce TNF-α-induced apoptosis. More importantly, AZD5582 induced a decrease of Mcl-1 protein, a member of the Bcl-2 family, but not that of Bcl-2 and Bcl-xL. Interestingly, ectopically expressing XIAP and cIAP1 inhibited the AZD5582-induced decrease of Mcl-1 protein, which suggests that AZD5582 elicits Mcl-1 decrease for apoptosis induction by targeting of XIAP and cIAP1. Taken together, these results indicate that sensitivity to AZD5582 is determined by p-Akt-inducible XIAP phosphorylation and by targeting cIAP1. Furthermore, Mcl-1 in pancreatic cancer may act as a potent marker to analyze the therapeutic effects of AZD5582.

## INTRODUCTION

The mortality rate of pancreatic carcinomas, which have a 5-year survival rate of less than 5%, is among the highest of all human cancer types [1]. Resistance of pancreatic cancers to even aggressive treatment regimens presents a major challenge in oncology. An underlying feature of this refractoriness to treatment, and a contributor to the aggressive nature of the disease, is apoptosis resistance. Previous studies in pancreatic cancer have described multiple defects in apoptosis signaling at different levels of the pathway [2]. Pancreatic cancer cells gain protection against the mitochondrial-dependent apoptotic pathway through overexpression of inhibitor of apoptosis proteins (IAPs), which are inhibitors of caspases [3], as well as through dysregulation of other apoptosis-modulating genes [4]. Understanding these mechanisms, which might have great potential as targets for overcoming therapy-resistance of human pancreatic cancer, has opened novel strategies for anticancer drug development.

IAPs appear to be essential for regulating the apoptotic program. IAPs contain one to three baculovirus IAP repeat (BIR) domains and one RING finger domain, the latter of which possesses E3 ligase activity [5]. The BIR domains of XIAP, cIAP1, and cIAP2 directly bind, and subsequently inhibit, caspases [6]. This inhibition can be abolished by IAP antagonists, such as Smac/Diablo [7, 8]. The C-terminal RING finger domain of IAPs regulates a number of protein targets by promoting their ubiquitination and proteasome-dependent degradation [9]. Previous studies have shown that IAPs are overexpressed in various cancers, including pancreatic cancer [3, 10, 11] and have implicated IAPs in tumor cell survival [12].

Natural IAP antagonists, including Grim and Smac/Diablo, have been shown to bind to the same groove in the BIR domains of IAPs as inhibitors of caspases [13]. Synthetic IAP antagonist compounds that mimic natural IAP antagonists have been designed based on this inhibitory mechanism. Such mimetic IAP antagonists have been developed using a variety of approaches, including large-scale combinational library screening [14]. These compounds have been shown to enhance the efficacy of chemotherapy and suppress tumor cell survival [15, 16]. To date, other groups are reported that IAP antagonist or SMAC minetic are potentiated anti-tumor effects in human pancreatic cancer cell lines [17–19]. These studies have evaluated the efficacy of IAP antagonists with chemo-agents against pancreatic cancer. However, no studies have demonstrated the sensitive or resistant mechanisms of IAP antagonists against pancreatic cancer cell lines. Moreover, the mechanism of action for IAP antagonists and the processes involved in resistance to these agents have not been widely investigated.

Here, we demonstrate that AZD5582, a synthetic small molecule and member of the IAP antagonist family [20], induces apoptosis in human pancreatic cancer cells by targeting XIAP and cIAP1. Our results also reveal the mechanism underlying the resistance to synthetic IAP antagonist, AZD5582.

## RESULTS

### Human pancreatic cancer cells display different sensitivities to the synthetic IAP antagonist, AZD5582

**Figure 1:**
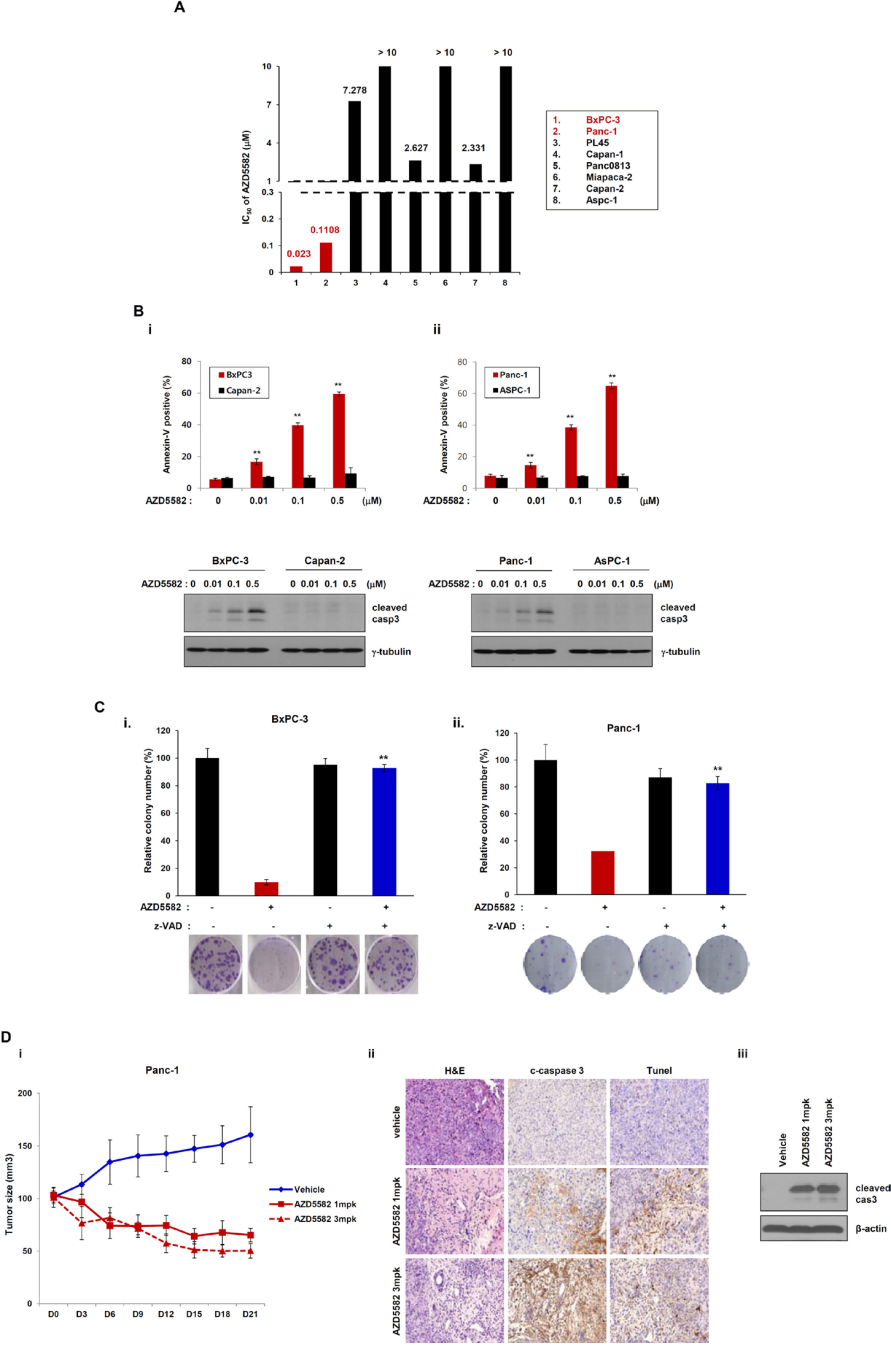
Human pancreatic cancer cells display different chemosensitivities to the IAP antagonist, AZD5582 **A.** Pancreatic cancer cell lines were treated with AZD5582 (39 nM −10 uM) for 72 h. After incubating for 72 h, MTS assays were performed. IC_50_ values are shown as the mean ± SE from three independent experiments performed in duplicate. **B.** BxPC3, Panc-1, AsPC-1 and Capan-2 were treated with the indicated doses of AZD5582 for 24 h and cell lysates were then analyzed by western blot using antibodies against cleaved caspase-3 and γ-tubulin. γ-tubulin was used as a loading control. AZD5582 differentially induces cell death in pancreatic cancer cells. BxPC-3, Capan-2 (left panel), Panc-1 and AsPC-1 cells (right panel) were treated with AZD5582 at the indicated doses for 24 h. These cells were harvested and stained using annexin V/PI. These experiments were analyzed by flow cytometry. The data are the means ± s.d. **P < 0.01. **C.** Colony-forming assays were performed on BxPC3 (left panel) and Panc-1 (right panel). The cells were treated with 100 nM AZD5582 in the presence or absence of z-VAD-FMK (pan-caspase inhibitor). After 24 h, the cells were harvested, counted, and seeded into 6-well plates at a density of 3 × 10^2^ cells/well. After 10–14 days the cells were fixed, stained, and photographed. The graphs present the relative number of colonies as the means ± SDs from three separate experiments performed in triplicate. **P < 0.01. **D.** Panc-1-derived xenograft model were treated with AZD5582. The tumor growth and weight were decreased by AZD5582. The expression of cleaved caspase 3 was increased by western blot analysis and immunohistochemistry. γ-tubulin was used as a loading control.

To validate the effect of AZD5582, we first examined its inhibitory effect on multiple human pancreatic cancer cell lines as a single agent. Differences were observed in the sensitivities of pancreatic cancer cell lines to AZD5582. BxPC-3 and PanC-1 cells were sensitive to the compound (Figure 1A and 1B), exhibiting IC_50_ values for AZD5582 of 23 nM and 110.8 nM, respectively. In contrast, all other cells exhibited resistance to AZD5582, with the exception of both cell types mentioned above (Figure 1A). We determined resistance cut-off value based on IC50 for AZD5582. Resistance cell lines exhibit pXIAP and pAKT expression. Panc08.13 and Capan-2 had 2uM IC50 for AZD5582 and exhibited pXIAP and pAKT. Thus, these cell lines can be considered similar to other resistance cell lines. Therefore, we can assume that BxPC-3 and Panc-1 cell lines are sensitive and that other cell lines are resistant to AZD5582. We next examined the inhibitory effect of AZD5582 on BxPC-3 and Panc-1 cells that were sensitive to AZD5582. AZD5582-induced apoptosis was measured the percentage of annexin V/PI-positive cells using flow cytometry. (Figure 1B, upper panel). AZD5582 induced the cleavage of caspase-3, resulting in active caspase-3, but only in the BxPC-3 & PanC-1 cell lines which were sensitive to AZD5582 (Figure 1B, lower panel), indicating that pancreatic cancer cells have differential sensitivity to AZD5582. Colony formation in both cell types significantly decreased after treatment with AZD5582 (Figure 1C), but not in cells that were resistant to AZD5582 (Supplementary Figure S1). In addition, we investigated whether AZD5582 inhibited tumor growth in Panc-1-derived xenograft model. After AZD5582 treatment, tumor growth and weight decreased, whereas cleaved caspase 3 expression increased. We analyzed apoptotic protein by Tunel assay (Figure 1D).

### Phospho-AKT-inducible XIAP phosphorylation induces resistance to AZD5582

**Figure 2:**
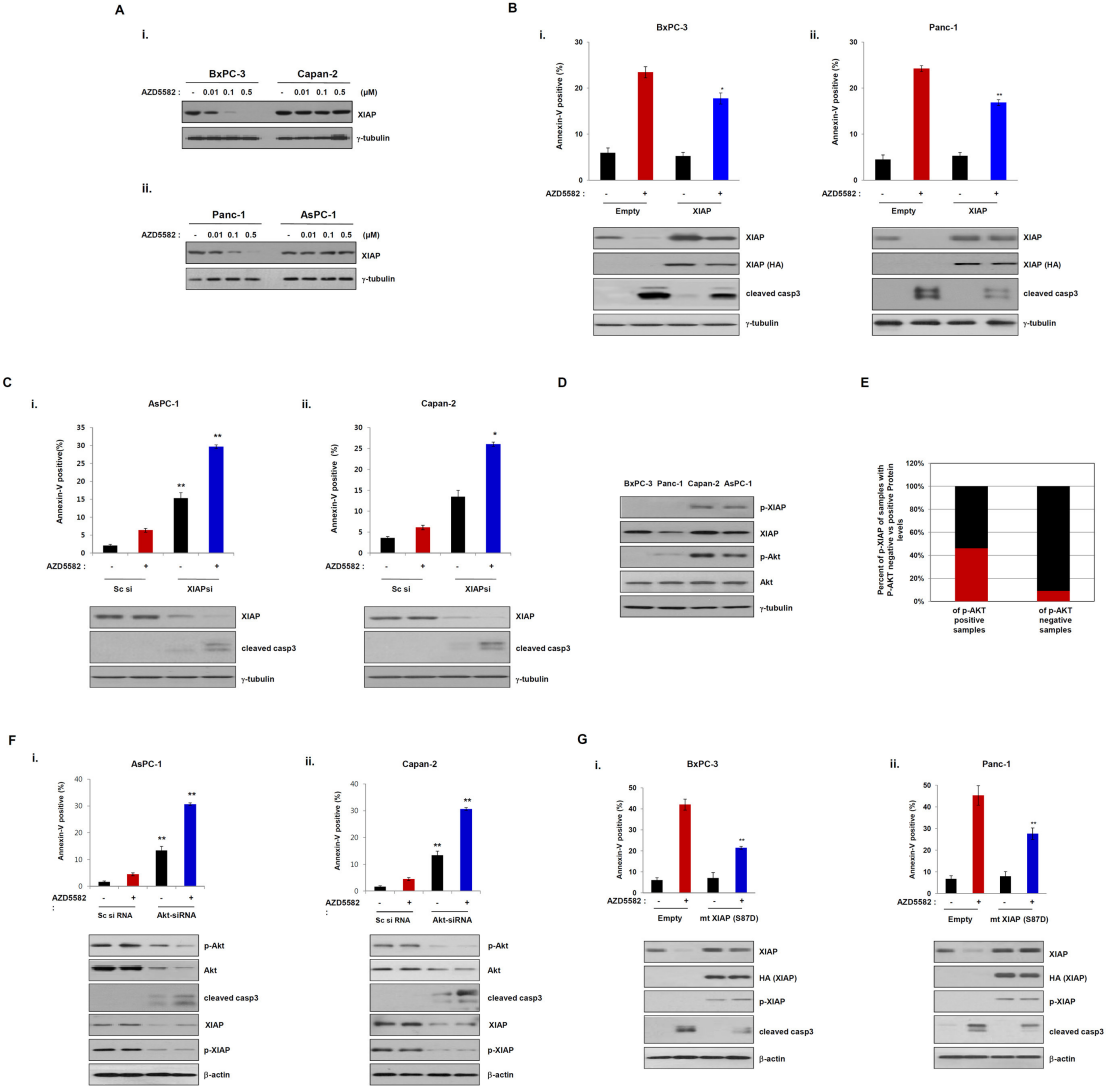
Phosphorylation of XIAP induces resistance to the IAP antagonist, AZD5582 **A.** BxPC-3, Capan-2 (upper panel), Panc-1 and AsPC-1 (lower panel) were treated with the indicated doses of AZD5582 and the cell lysates were then immunoblotted using XIAP and γ-tubulin antibodies. γ-tubulin was used as a loading control. **B.** BxPC-3 (left panel) and Panc-1 (right panel) cells were transfectd with empty or XIAP cDNA for 24 h and incubated with 100 nM AZD5582 another 24 h. The cells were trypsinized, washed with PBS, incubated annexin-V staining solution (BD Pharmingen) and then analyzed with flow cytometry. Cell lysates were analyzed by immunoblot using antibodies against XIAP, HA, cleaved caspase 3 and γ-tubulin. γ-tubulin was used as a loading control. The values are presented as the means ± SDs from three separate experiments performed in triplicate. *P < 0.05, **P < 0.01. **C.** AsPC-1 and Capan-2 cells were transfected with scramble siRNA or XIAP siRNA for 24 h and then treated with 100 nM AZD5582 for 24 h. Annexin-V positive cells were analyzed as described 2B. Cell lysates were immunoblotted using antibodies against XIAP, cleaved caspase 3 and γ-tubulin. γ-tubulin was used as a loading control. The values are presented as the means ± SDs from three separate experiments performed in triplicate. *P < 0.05, **P < 0.01. **D.** Basal levels of phospho-Akt and phospho-XIAP in four pancreatic cancer cell lines were determined by immunoblot analysis. **E.** The graph presents the correlation between phosphorylation of Akt and XIAP in pancreatic cancer tissues (*n* = 24). **F.** AsPC-1 and Capan-2 cells were transfected with Sc siRNA or AKT siRNA for 24 h and then treated with 100 nM AZD5582 another 24 h. The population of annexin-V positive cells was performed according to 2B. Cell lysates were analyzed by immunoblot using antibodies against phospho-Akt, Akt, cleaved caspase-3, XIAP, phospho-XIAP, and γ-tubulin. γ-tubulin was as a loading control. The values were represented as the means ± SDs from three separate experiments performed in triplicate. **P < 0.01. **G.** BxPC3 and Panc1 cells were transfected with empty expressing plasmid or XIAP S87D expressing plasmid for 24 h and then incubated with 100 nM AZD5582 for 24 h. Annexin-V positive cells were determined by flow cytometry. Cell lysates were analyzed by immunoblot using antibodies against XIAP, HA, phospho-XIAP, cleaved caspase-3 and γ-tubulin. γ-tubulin was used as a loading control. The values are presented as the means ± SDs from three separate experiments performed in triplicate. *P < 0.05, **P < 0.01.

As shown in Figure 1, the human pancreatic cancer cell lines tested displayed different sensitivities to AZD5582, with Capan-2 and AsPC-1 cells displaying resistance to AZD5582. Consistently, the cleavage of caspase-3 was observed in AZD5582-sensitive cells, but not in AZD5582-resistant cells. Based on a report demonstrating that XIAP directly inhibits active caspase-3 [21], we investigated the inhibitory effect of AZD5582 on XIAP. XIAP expression was significantly decreased after exposure to AZD5582 in BxPC-3 and PanC-1 cells that are sensitive to AZD5582, but not in Capan-2 and AsPC-1 cells that are resistant to AZD5582 (Figure 2A). To further analyze whether the difference in sensitivity to AZD5582 is dependent on XIAP, we first selected the two pancreatic cancer cells, BxPC-3 and PanC-1, sensitive to AZD5582. BxPC-3 and PanC-1 cells were transfected with a construct expressing XIAP cDNA, or a control vector, followed by AZD5582 treatment. Cells expressing ectopic XIAP displayed decreased sensitivity to AZD5582 (Figure 2B and Supplementary Figure S2A). However, transfection with XIAP did not completely inhibit the cleavage of caspase-3 after treatment with AZD5582. Next, we examined the effects of XIAP silencing via small interfering RNA (siRNA) on two pancreatic cancer cell lines, Capan-2 and AsPC-1, which are resistant to AZD5582. XIAP knockdown resulted in increased cell death in both cell types after exposure to AZD5582 (Figure 2C and Supplementary Figure S2B). These results suggested that AZD5582 induces apoptotic cell death through the inhibition of XIAP in pancreatic cancer cells.

It has previously been reported that XIAP phosphorylation by AKT protects XIAP from ubiquitination and degradation in response to apoptotic stress [22]. Thus, to investigate the mechanism underlying the resistance to AZD5582, we first examined the basal levels of phospho-AKT and phospho-XIAP in the four pancreatic cancer cell lines. Consistent with a role for the previously reported AKT-dependent resistance mechanism, we found that both AKT and XIAP were phosphorylated in resistant Capan-2 and AsPC-1 cells, but not in sensitive BxPC-3 and Panc-1 cells (Figure 2D), suggesting that the phosphorylation of XIAP by phospho-AKT induces resistance to AZD5582. In view of these results, we evaluated the levels of phospho-XIAP and phospho-AKT in human pancreatic normal and cancer tissues. Interestingly, phosphorylated XIAP was found in 7 of 24 human pancreatic cancer tissues, but was almost undetectable in normal tissues. Furthermore, we observed a significant relationship between phospho-AKT and phospho-XIAP expression in tissue samples from patients with pancreatic cancer. However, the expression of the other phosphorylated proteins, with the exception of phospho-XIAP expression, was detected in normal and cancer tissues (Figure 2E and Supplementary Figure S3). This finding supports the potential value of AZD5582 therapy for pancreatic cancer patients; however, prospective patients should be evaluated for their phospho-XIAP status.

To confirm that the phosphorylation of AKT induces resistance to AZD5582, we examined the effects of AKT silencing via small interfering RNA (siRNA) on two pancreatic cancer cell lines, Capan-2 and AsPC-1. AKT knockdown caused a decrease of phospho-XIAP levels (Figure 2F). Consistently, the apoptotic-cell population significantly increased in cells expressing AKT-siRNA followed by AZD5582 treatment (Figure 2F and Supplementary Figure S2C). To further study the relationship between XIAP phosphorylation and resistance to AZD5582, we constructed a mutant XIAP (HA-tagged XIAP-S87D) in which Ser-87 was replaced with aspartic acid. As expected, apoptotic cell death decreased in BxPC-3 and PanC-1 cells transfected with the mutant XIAP (HA-tagged XIAP-S87D) following AZD5582 treatment (Figure 2G and Supplementary Figure S2D). These results suggest that the phosphorylation of XIAP by AKT induces resistance to AZD5582, and further indicates that the phosphorylation of both AKT and XIAP in human pancreatic cancer may be a predictive biomarker for the efficacy of AZD5582 treatment.

### The synthetic IAP antagonist AZD5582 decreases growth of tumors bearing ASPC-1 and Capan-2 that stably express AKT-shRNA

**Figure 3:**
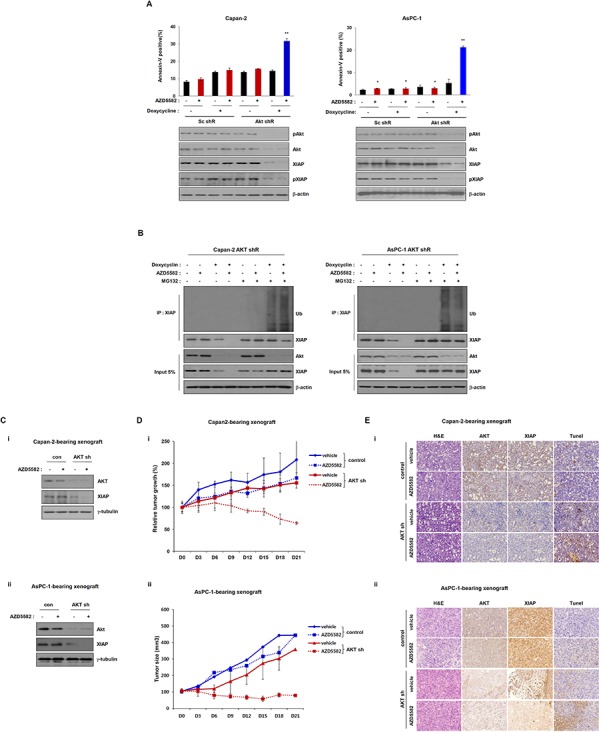
Tumor growth in the Capan-2 and AsPC-1 inducible AKT-shRNA-bearing xenograft model is inhibited by AZD5582 **A.** Doxycycline-inducible AKT shRNA Capan-2 and AsPC-1 cells were treated with 1 μg/ml doxycycline in the presence/absence of AZD5582 (100 nM). These cells were harvested and analyzed annexin-V positive cells by flow cytometry. Cell lysates were used for immunoblotting using Akt, pAkt, XIAP and pXIAP antibodies, and β-actin was used as an internal loading control. **B.** Capan-2 (left panel) and AsPC-1 (right panel) stable AKT-shRNA were treated with 100 nM AZD5582 in the presence or absence of doxycycline (1 μg/ml). Ubiquitination of XIAP was verified by immunoprecipitation using anti-XIAP antibody. Cells were analyzed by immunoblot analysis using antibodies against ubiquitin, phospho-Akt, Akt, XIAP, phospho-XIAP, and β-actin. β-actin was used as a loading control. The values are presented as the means ± SDs from three separate experiments performed in triplicate. **P < 0.01. **C.** Immunoblot analysis of tumors derived from inducible Akt shRNA-transfected Capan-2 (i) and AsPC-1 (ii) showing that Akt expression is decreased after treatment with doxycycline. And XIAP expression is decreased after treatment with doxycycline. **D.** Growth of tumors derived from inducible Akt shRNA-transfected Capan-2 (i) and ASPC-1 (ii) was reduced with AZD5582 (3 mg/kg) in the presence of doxycycline (10 mg/kg). These data are presented as the means ± SEM. **P < 0.01. **E.** Immuohistochemical analysis demonstrated that the expression of XIAP and AKT were decreased after treatment with doxycycline. Additionally, apoptotic protein was detected by Tunel assay.

Because AKT positively regulates XIAP protein stability by inhibiting its degradation, and AKT knockdown resulted in XIAP degradation, as described above. We posited that AKT might function as a resistance factor against AZD5582 by stabilizing XIAP. To test this possibility, we examined the inhibitory effect of AKT knockdown on the induction of cell death after exposure to AZD5582. We first established derivatives of Capan-2 and AsPC-1 cell lines that stably express doxycycline-inducible AKT-shRNA vectors. Treatment with doxycycline clearly led to decreased XIAP expression and phosphorylation in Capan-2 and AsPC-1 cells that express doxycycline-inducible AKT-shRNA vectors (Figure 3A). Conversely, apoptotic cell death in Capan-2 and AsPC-1 derivatives was significantly increased after exposure to doxycycline, but not in derivatives that were not treated with doxycycline (Figure 3A and Supplementary Figure S4). Additionally, AKT can induce resistance to AZD5582 by stabilizing the XIAP protein. We next investigated the effects of AKT on AZD5582-induced XIAP ubiquitination. Capan-2 and AsPC-1 AKT shRNA inducible cells were treated with doxycycline and AZD5582 in the presence/absence of MG132. Ubiquitination of XIAP was analyzed by immunoprecipitation and immunoblotting after 24 h of the treatment. As shown in Figure 3B, AZD5582 induces ubiquitination of XIAP that was significantly inhibited by AKT expression (Figure 3B). Taken together, we conclude that AKT can induce resistance to AZD5582 by stabilizing the XIAP protein.

To further examine the inhibitory effect of AZD5582 on Capan-2 and AsPC-1 derivative cell lines, we established each derivative-bearing mouse xenografts with a doxycycline-inducible stable depletion of AKT. Cells from each derivative were subcutaneously injected into mice and allowed to form xenograft tumors, and they were then treated with doxycycline or vehicle. Tumors derived from derivative-inducible, stable clones of Capan-2 and AsPC-1 derivatives showed decreased AKT levels after an injection of doxycycline (Figure 3C). Tumor growth in doxycycline-treated mice was slightly inhibited, because AKT expression was decreased. In addition, tumor growth in doxycyclin-treated mice was clearly decreased compared with that in control mice treated with vehicle alone in the presence of AZD5582. However, tumor growth was not changed, although we administrated AZD5582 in the Capan-2 and AsPC-1-derived xenograft models (Figure 3D). Next, we analyzed AKT and XIAP expression using immunohistochemistry and detected apoptotic protein by Tunel assay. Expression of these proteins was decreased in tissues from doxycycline-treated mice from the Capan-2 and AsPC-1 derivative-xenograft (Figure 3E). Consistently, western blot analysis also showed decreases of these protein levels (Figure 3C), indicating that AZD5582 can suppress tumor growth in combination with AKT inhibition.

### AZD5582 induces apoptotic cell death through TNFα-dependent cIAP1 degradation

**Figure 4:**
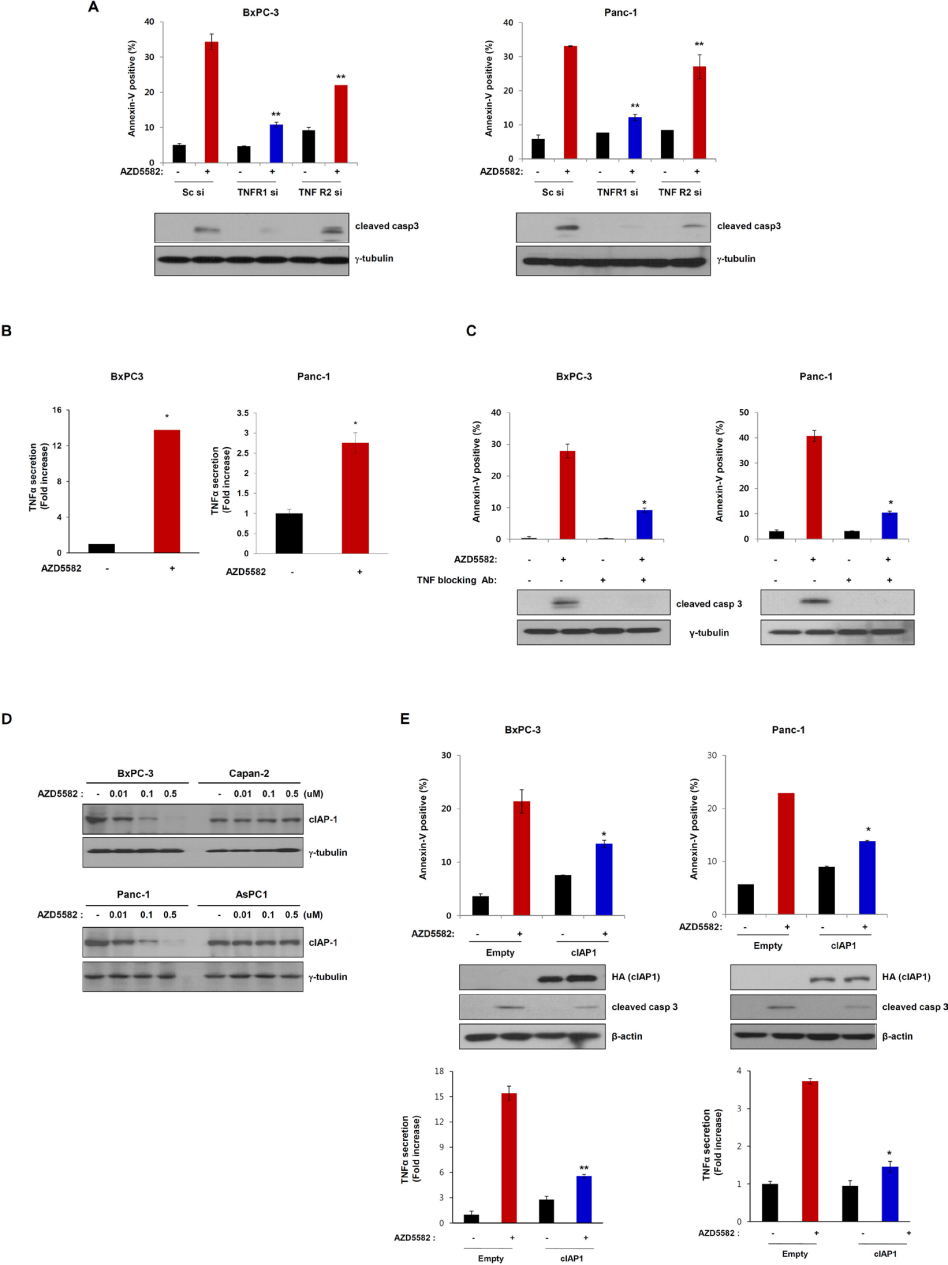
AZD5582 induces cell death through TNF-α-dependent apoptosis **A.** BxPC-3 and Panc-1 cells were transfected with Sc siRNA or TNFR1 siRNA or TNFR2 siRNA for 24 h and then treated with 100 nM AZD5582 for 24 h. These cells were stained with annexin-V and analyzed by flow cytometry. Cell lysates were prepared for immunoblot analysis against anti-caspase-3 and the loading control γ-tubulin. The error bars represent the mean ± s.d. of three separate experiments performed in triplicate. **P < 0.01. **B.** BxPC-3 and Panc-1 cells were treated with 100 nM AZD5582 for 24 h then cell supernatants were collected for TNF-α ELISA assays. The graph displays the means ± s.d. **P < 0.01. **C.** BxPC-3 and Panc-1 cells were incubated with 5 μg/ml TNF-α blocking antibodies in the presence/absence of AZD5582. Annexin-V positive cells were analyzed using flow cytometry. Cells were treated with 100 nM AZD5582 and TNF-α blocking antibodies at 5 μg/ml. The expression of cleaved caspase-3 was verified by immunoblot analysis with anti-caspase-3 antibody. γ-tubulin was used as a loading control. Data are the means ± s.d. **P < 0.05. **D.** Cells were treated with AZD5582 at the indicated doses for 24 h. Cell lysates were subjected to immunoblot analysis using anti-cIAP1 and γ-tubulin antibodies. **E.** BxPC-3 and Panc-1 cells were transfected with empty or cIAP1 expressing plasmid for 24 h and the cells were treated with 100 nM AZD5582 for 24 h. Annexin-V positive staining was measured by flow cytometry. These cells were harvested and analyzed by immunoblotting against anti-HA, anti-caspase-3 and the loading control γ-tubulin. Cell supernatants were collected and analyzed by TNF-α ELISA assays. *P < 0.05.

It has been reported that IAP antagonists inhibit cIAP1 to induce TNFα-dependent apoptosis [16]. We therefore analyzed the involvement of TNF-R in AZD5582-induced apoptotic cell death. We first examined the knockdown effect of TNFR1- and TNFR2-siRNA on BxPC-3 and PanC-1 cells which are sensitive to AZD5582. The number of dead cells decreased in TNFR1, and TNFR2-siRNA-treated BxPC-3 or PanC-1 cells following AZD5582 treatment (Figure 4A and Supplementary Figure S5). In particular, TNFR1 knockdown resulted in less cell death compared with TNFR2-knockdown, implying that AZD5582 may induce cell death via the TNFR signaling pathway. To confirm whether AZD5582 induced TNFR-related apoptotic cell death, we first examined TNFα production after exposure to AZD5582. Treatment with AZD5582 resulted in TNFα production in BxPC-3 and PanC-1 cells which are sensitive to AZD5582 (Figure 4B). In contrast, resistance cell lines did not produce TNFα under AZD5582 treatment (Supplementary Figure S6). To next determine whether cell death induced by AZD5582 required TNFα, we incubated AZD5582-sensitive cells with AZD5582 in the presence or absence of antibodies that block TNFα. Consistent with a requirement for TNFα, AZD5582-induced cell death was dramatically inhibited after exposure to TNFα-blocking antibodies (Figure 4C and Supplementary Figure S7A), suggesting that TNFα production elicited by AZD5582 can induce apoptotic cell death in human pancreatic cancer cells.

TNFα production induced by AZD5582 was significantly correlated with the induction of apoptosis. Recently, it has been reported that IAP antagonists target cIAP1 to induce TNFα-dependent apoptosis. Accordingly, we investigated whether AZD5582 targets cIAP1 in our system. Treatment with AZD5582 resulted in a decreased level of cIAP1 in BxPC-3 and PanC-1 cells which are sensitive to AZD5582, but not in Capan-2 and AsPC-1 cells, which are resistant to AZD5582 (Figure 4D). To further confirm the involvement of cIAP1 in AZD5582-induced cell death, we transfected cells with a construct expressing cIAP1 cDNA following AZD5582 treatment. The expression of cIAP1 inhibited AZD5582-induced cell death and TNFα production (Figure 4E and Supplementary Figure S7B), indicating that AZD5582 targets cIAP1 in human pancreatic cancer cells.

### AZD5582-induced cell death can be completely blocked by the inhibition of both XIAP and cIAP1

**Figure 5:**
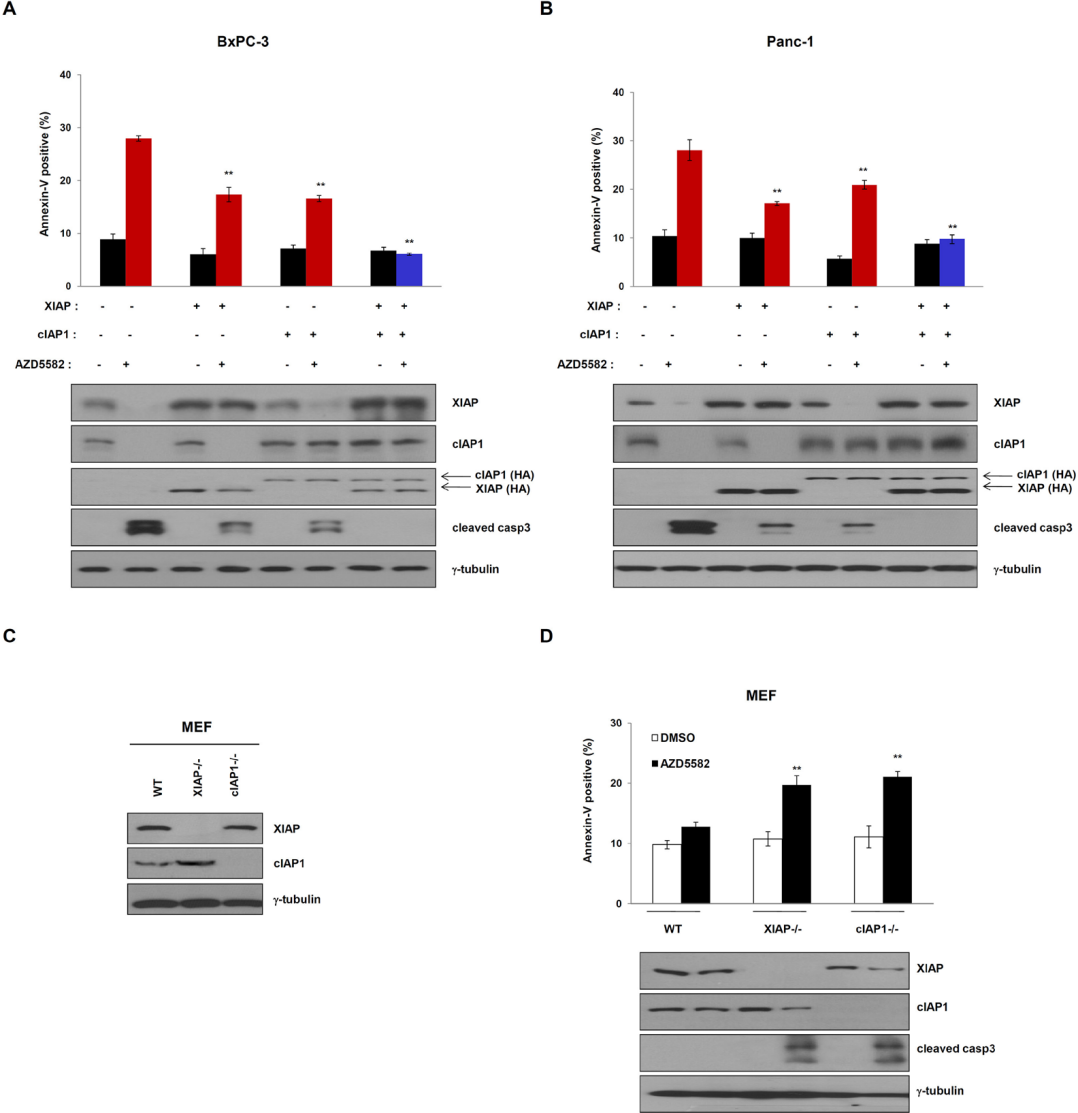
Overexpression of XIAP and cIAP1 completely inhibits AZD5582-induced cell death HA-tagged XIAP and/or cIAP1 vector transfected **A.** BxPC-3 and **B.** Panc-1 cells were transfected with XIAP and/or cIAP1 expressing plasmid for 24 h, treated with 100 nM AZD5582 for 24 h. These cells were analyzed annexin-V staining and immunoblotting against XIAP, cIAP1, HA, cleaved caspase-3. γ-tubulin as a loading control. The data are the means ± s.d. **P < 0.01, *P < 0.05 **C.** Expression of XIAP and cIAP1 in XIAP-null or cIAP1-null mouse embryonic fibroblasts (MEF). Wild-type, XIAP null and cIAP1 null MEFs were subjected to immunoblot analysis with anti-XIAP and anti-cIAP1 antibodies. γ-tubulin was used as a loading control. **D.** Wild type, XIAP-null and cIAP1-null mouse embryonic fibroblast cells were treated with optimal doses of AZD5582. Annexin-V positive cells were analyzed by flow cytometry. **P < 0.01. Immunoblot analysis was performed with anti-XIAP, anti-cIAP1 and cleaved caspase-3 antibodies. γ-tubulin was used as a loading control. The data are the means ± s.d. **P < 0.01, *P < 0.05

As shown in Figure 2 and Figure 4, the expression of XIAP or cIAP1 partially inhibited AZD5582-induced cell death, respectively. We therefore tested whether the expression of both XIAP and cIAP1 fully inhibits AZD5582-induced cell death. To do this, we transfected cells with constructs expressing XIAP and/or cIAP1 cDNA, followed by AZD5582 treatment. BxPC-3 cells, displaying sensitivities to AZD5582 and expressing both XIAP and cIAP1 were more resistant to AZD5582 than those transfected with either XIAP or cIAP1 (Figure 5A and Supplementary Figure S8A, i). Consistently, apoptotic-cell populations in PanC-1 cells expressing both cDNA clearly decreased after exposure to AZD5582 (Figure 5B and Supplementary Figure S8A, ii). To further examine this, we used wild-type, XIAP-knockout and cIAP1-knockout MEFs (Figure 5C). Wild-type MEFs were insensitive to AZD5582, whereas the cell death rate in XIAP-knockout or cIAP1-knockout MEFs was significantly increased after exposure to AZD5582 (Figure 5D and Supplementary Figure S8B). Consistent with these results, cIAP1 levels in XIAP-knockout, or XIAP levels in cIAP1-knockout MEFs was decreased in response to AZD5582, suggesting that the inhibition of both XIAP and cIAP1 is required to kill these AZD5582-sensitive pancreatic cancer cells.

### Overexpression and stabilization of Mcl-1 renders resistance to AZD5582

**Figure 6:**
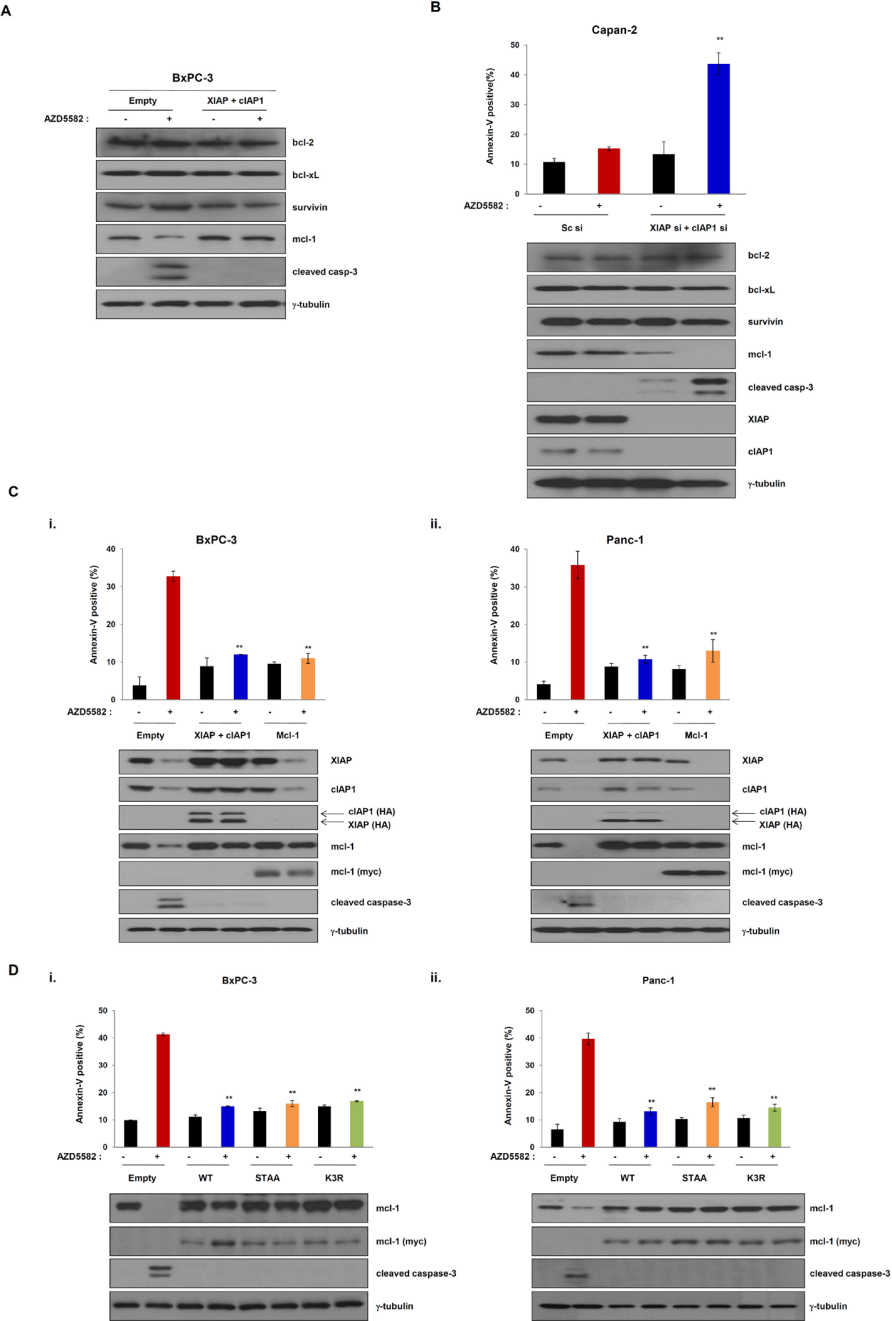
Overexpression of Mcl-1 mediates resistance of the IAP antagonist AZD5582 **A.** The expression of Mcl-1 was verified by immunoblot analysis using cell lysates which were co-transfected with XIAP- or cIAP1-expressing constructs for 24 h, and treated with 100 nM AZD5582 for 24 h. Immunoblot analysis was performed using anti-bcl-2, anti-bcl-xL, anti-survivin, anti-mcl-1 and anti-cleaved caspase-3 antibodies. γ-tubulin was used as a loading control. **B.** Capan-2 cells were transfected with scramble siRNA or XIAP and cIAP1 siRNA and treated with 100 nM AZD5582 for 24 h. These cells were stained annexin-V and analyzed by flow cytometry. And the cells were harvested and analyzed by immunoblotting against bcl-2, bcl-xL, survivin, mcl-1, cleaved caspase-3, XIAP and cIAP1 antibodies. γ-tubulin was used as a loading control. Data are the means ± s.d. **P < 0.01, *P < 0.05. **C.** BxPC-3 (i) and Panc-1 (ii) cells were transfected with empty or XIAP and cIAP1 or Mcl-1 expressing plasmid for 24 h and then incubated with 100 nM AZD5582 for 24 h. Population of apoptotic cells was stained annexin-V solution and analyzed by flow cytometry. (upper panel). Cell lysates were prepared for immunoblot analysis using anti-XIAP, anti-cIAP1, anti-HA, anti-mcl-1, anti-myc and cleaved caspase 3 antibodies. γ-tubulin was used as a loading control. Data are the means ± s.d. **P < 0.01, *P < 0.05 **D.** BxPC-3 (i) and Panc-1 (ii) cells were transfected with Mcl-1 wild type or STAA (Ser168 and Thr163 substituted with alanine) or K3R (K136, K194 and K197 substituted with arginine) for 24 h then treated with 100 nM AZD5582 for 24 h. The cells were stained with annexin-V solution and analyzed by flow cytometry. (upper panel). The expression levels of Mcl-1 and cleaved caspase-3 were determined by immunoblot analysis against mcl-1, myc, cleaved caspase-3 and γ-tubulin. The graphs are the means ± s.d. **P < 0.005.

As demonstrated by the above results, AZD5582 induces cell death by targeting XIAP and cIAP1. We therefore investigated whether AZD5582 affects other anti-apoptotic proteins, such as Bcl-2 family members (Bcl-2, Bcl-xL, and Mcl-1), or survivin. We first observed a change of these protein levels in cells expressing both XIAP and cIAP1, or in cells expressing an empty vector after exposure to AZD5582. Surprisingly, treatment with AZD5582 resulted in a decrease of Mcl-1 levels in cells expressing the control vector, whereas the level of Bcl-2, Bcl-xL, or survivin was not changed (Figure 6A). Furthermore, Mcl-1 levels in cells expressing XIAP and cIAP1 were not affected by AZD5582 (Figure 6A). Next, we analyzed the effect of AZD5582 on these anti-apoptotic proteins using XIAP or cIAP1 silencing via small interfering RNA (siRNA). The knockdown of both XIAP and cIAP1 resulted in a decrease of Mcl-1 protein levels, whereas the levels of Bcl-2, Bcl-xL, or survivin were not decreased (Figure 6B). The cell death rate dramatically increased in XIAP- and cIAP1-siRNA-treated Capan-2 cells that are resistant to AZD5582 (Figure 6B and Supplementary Figure S9A), suggesting that Mcl-1 inhibition is required for AZD5582-induced cell death.

To further analyze whether AZD5582-induced cell death is dependent on Mcl-1 protein, we transfected AZD5582-sensitive cells with a construct expressing Mcl-1 cDNA following AZD5582 treatment. The expression of Mcl-1 confers resistance to AZD5582, similarly to the effects of XIAP and cIAP1 expression (Figure 6C and Supplementary Figure S9B). Consistently, the cleavage of caspase-3 was not observed in cells expressing Mcl-1 after exposure to AZD5582. Next, we further confirmed the involvement of Mcl-1 in AZD5582-induced cell death. Based on reports [23–25], Mcl-1 is a protein that experiences rapid turnover and is quickly degraded upon a variety of apoptosis-inducing signals (Supplementary Figure S10). In contrast, mutant (constitutive active form) Mcl-1 is resistant to apoptosis-inducing agents. Thus, we demonstrated that Mcl-1 mutants overcome AZD5582-induced apoptosis. On the other hand, no investigation has reported on the inactive non-phosphorylated form of Mcl-1. To do this, we first constructed wild-type Mcl-1 and constitutively active mutants-Mcl-1 (Myc-tagged Mcl-1-STAA and -K3R), in which alanine was substituted for phosphorylated amino acid sites in the domain. Then, we examined whether wild-type Mcl-1, -STAA or -K3R renders AZD5582-sensitive BxPC3 cells and PanC-1 cells resistant to AZD5582 by comparing the apoptotic-cell populations in AZD5582-treated BxPC3 and PanC-1 cells expressing wild-type Mcl-1, -STAA or -K3R with those in cells transfected with an empty vector. Cells expressing wild-type Mcl-1, -STAA or -K3R were more resistant to AZD5582 than those transfected with an empty vector (Figure 6D and Supplementary Figure S9C), indicating that the expression of Mcl-1 and the stabilization induced by mutating the domain within it renders cells resistant to AZD5582

## DISCUSSION

Because pancreatic cancer exhibits resistance to apoptosis inducers, such as gemcitabine, chemotherapeutic options are limited, and such agents only minimally enhance survival [1]. Therapies that increase apoptosis, an event that is critically important for the inhibition of tumorigenesis, are highly attractive for treatment of pancreatic cancers as well as other solid tumors. Among the cellular factors that play a key role in regulating apoptosis are IAPs, which, studies have shown, are pathologically overexpressed in pancreatic cancer [3]. These observations motivated our exploration of IAP-inhibition as a therapeutic strategy in pancreatic cancer. In this study, we demonstrated the inhibitory effect of the novel synthetic IAP antagonist, AZD5582, on pancreatic cancer cells. Our *in vitro* data showed that Panc-1 and BxPC-3 cells were sensitive to AZD5582, and experiments using a xenograft mouse model showed that AZD5582 was effective against tumors of Capan-2 or AsPC-1 derivative-xenografts *in vivo*.

AZD5582 did not induce a decrease in IAP levels in Capan-2 or AsPC-1 cells, which exhibited resistance to apoptosis. It was recently reported that phospho-AKT induces phosphorylation at Ser87 in the N-terminus of XIAP, resulting in XIAP stabilization [22]. Interestingly, our data also demonstrated that the phosphorylation of AKT and XIAP in the AZD5582-resistant cell lines Capan-2 and AsPC-1, but not in AZD5582-sensitive BxPC-3 and Panc-1 cells. The exogenous expression of XIAP S87D resulted in resistance to AZD5582 in AZD5582-sensitive BxPC-3 and Panc-1 cells, suggesting that the phosphorylation of XIAP renders cells resistant to AZD5582, suggesting that AZD5582 can suppress growth of tumors in combination with AKT inhibition.

Importantly, cIAP1 was also degraded in BxPC-3 and Panc-1 cells after exposure to AZD5582. We demonstrated that AZD5582 interacted with cIAP1 by SPR (Surface plasmon resonance) analysis (Supplementary Figure S11). Unlike cIAP1, cIAP2 protein levels were not decreased in AZD5582-sensitive cell lines. No mutations known to affect the stability of IAP proteins were found in the RING domain of cIAP2 in the AZD5582- sensitive cell lines, BxPC-3 and Panc-1 (Supplementary Figure S12). Furthermore, AZD5582 significantly increased tumor necrosis factor-α (TNFα) production in AZD5582-sensitive BxPC3 cells (Figure 4), but not in AZD5582-resistant cells (Supplementary Figure S6). These results are in accord with previous reports that cells sensitive to Smac mimetics produce or can be induced to produce more TNFα after exposure to a Smac mimetic [16]. Therefore, these findings indicate that the novel synthetic IAP antagonist, AZD5582, induces apoptosis in pancreatic cancer cells. However, the relationship between AZD5582 and cIAP1 to protein stability in AZD5582-resistant cells remains to be elucidated.

Collectively, these data suggest that XIAP phosphorylation can be used as a predictive biomarker for the efficacy of AZD5582 in pancreatic cancer treatment, and the use of therapeutic agents that interfere with IAP expression or function, such as AZD5582, may be a promising approach for the treatment of pancreatic cancer and are attractive as adjuvants to conventional chemotherapy.

## MATERIALS AND METHODS

### Cell culture and reagents

Synthetic IAP antagonist, AZD5582 was supplied by AstraZeneca. Co., Ltd. (Maccelsfiled, Cheshire, UK). The human pancreatic cancer cell lines including BxPC-3, Miapaca-2, Panc-1, Panc0813, PL45, Capan-1, Capan-2 and AsPC-1 were purchased from ATCC (American Type Culture Collection, Manassas, VA, USA). All cells were cultured according to the ATCC's recommended guide lines. XIAP and cIAP1 null mouse embryonic fibroblast cells were kindly provided by Dr. Colin S. Duckett (University of Michigan, Ann Arbor, MI, USA) and Dr. Robert Korneluk (Ontario Cancer Institute, USA), respectively. Cycloheximide, a protein synthesis inhibitor, MG132, a proteasome inhibitor and z-VAD, a pan-caspase inhibitor, were provided by Sigma (St. Louis, MO, USA).

### Plasmids, siRNA and transfection

Plasmids encoding HA-tagged XIAP and cIAP1 were kindly provided by Dr. Colin S Duckett (University of Michigan, Ann Arbor, MI, USA). Mcl-1 cDNA was purchased from Origene (Rockville, MD, USA). XIAP^S87D^, Mcl-1^STAA^ and Mcl-1^K3R^ mutant constructs, in which Ser87 or Ser168, Thr163 or K136, K194, K197 were replaced with aspartic acid (XIAP^S87D^), alanine (Mcl-1^STAA^) or arginine (Mcl-1^K3R^), were constructed using the PCR-based Quik ChangeSite-Directed Mutagenesis Kit (Intron biotechnology, Seoul, Republic of Korea). Human pancreatic cancer cells were transiently transfected with scrambled (control) siRNA or siRNA against XIAP, Akt, cIAP1, TNFR1 or TNFR2 (150 pmol siRNA per 60 mm dish) using Lipofect-AMINE 2000 (Invitrogen, Carlsbad, CA, USA). The siRNAs used targeted the following specific sequences: scramble siRNA: 5′-GCG CAU UCC AGC UUA CGU A-3′, XIAP: 5′-GTA AAA TGC AAG TGG CAA ATT-3′, Akt: 5′-GGA CAA GGA CGG GCA CAU UU-3′, cIAP1: 5′-AAA GAG AGC CAU UCU GUU CUU-3′, TNFR1: 5′-CGG CAU UAU UGG AGU GAA A-3′, and TNFR2: 5′- CCG GGA AGC GAU GAA UUU GGA UU-3′. All siRNAs were obtained from Genolution Pharmaceuticals Inc. (Seoul, Republic of Korea).

### Cell death and MTS assay

DNA or siRNA-transfected or AZD5582-treated cells were collected and cell death was determined by trypan blue exclusion using at least 200∼500 cells. For the MTS assay, pancreatic cancer cells were seeded at 1∼3 × 10^4^ cells/well in a 96-well microtiter plate. The following day, cells were treated with AZD5582, an IAP antagonist, with various doses for 72 h. The MTS assay was performed according to the MTS assay protocol (Promega Inc, Madison, WI, USA). The IC_50_ of AZD5582 was determined using GraphPad Prism version 5.01 software.

### Doxycycline-inducible Akt shRNA stable cell lines

AsPC-1 and Capan-2 cells were co-transfected with pcDNA6.0/TR (Invitrogen) and H1-Akt shRNA-expressing plasmids and then selected by treating with blasticidin (5 μg/ml) and zeocin (150 μg/ml) for 3 weeks. To verify the Akt-inducible stable cell line, selected cells were treated with doxycycline for 48 h and then confirmed with immunoblot analysis for downregulating Akt using anti-Akt antibodies.

### Immunoblot analysis

Immunoblot analyses were performed by preparing cell lysates in RIPA lysis buffer containing protease- and phosphatase-inhibitor cocktails (Sigma). Protein concentrations were determined using the Bradford assay, and 20 μg of total cellular protein per sample were resolved by 8∼15% SDS-PAGE then transferred to an Immobilon-P membrane (EMD Millipore, Billerica, MA, USA). Membranes were blocked using 5% nonfat dry milk in TBS-T buffer (20 mM Tris-HCl, pH 7.4, 150 mM NaCl and 0.1% Tween 20) and probed with antibodies against HA, Akt, γ-tubulin (Santa Cruz Biotechnology, Santa Cruz, CA), cIAP1, XIAP, Mcl-1, myc, phospho-AKT, survivin, bcl-2, bcl-xL (Cell Signaling Technology, Beverly, MA, USA) or phosphor-XIAP (Abcam, Cambridge, MA, USA). Primary antibodies were detected using horseradish peroxidase-conjugated goat anti-mouse, goat anti-rabbit or donkey anti-goat secondary antibodies, as appropriate and visualized using enhanced chemiluminescence reagents (Amersham, Buckinghamshire, UK). Human normal and cancerous pancreatic tissues were obtained from pancreatic cancer patients after obtaining informed consent. The biospecimen and data used in this study was provided by Asan Bio-Resource Center.

### Annexin V or Annexin V/PI staining

The cells were harvested, washed with PBS and incubated with Annexin V binding buffer according to manufacturer's instructions. And then the cells stained with Annexin V or Annexin V/PI. Flow cytometry analyses performed on a FACS Calibur, Canto (BD Bioscience) or Muse (Millipore) instruments.

### *In vivo* tumor xenografts and immunohistochemistry

Xenograft tumor growth in Balb/c nude mice was used as a model for *in vivo* experiments. All animal studies were conducted in compliance with animal protocols approved by the Asan Medical Center Institutional Animal Care and Use Committee. Panc-1 and doxycycline-inducible Akt shRNA stable cell lines (Capan-2 and Aspc-1) were harvested and resuspended in matrigel at a concentration of 1 × 10^7^ or 5 × 10^6^ cells/100 μl, and the cells were then implanted subcutaneously. When tumor sizes reached 100 mm^3^, the mice were administered 10 mg/kg doxycycline (day 0), delivered in water, and the tumor volumes were monitored for 3 weeks. Mice were administered vehicle or the indicated concentrations of AZD5582 by i.v. once a week for three weeks, and the tumors were measured every 3 days for 3 weeks. Body weights were monitored every 3 days for 3 weeks. At 3 weeks, tumors were excised and fixed in formalin. XIAP and AKT were detected in tumor tissues by immunohistochemistry. Immunohistochemical analyses were performed as previously described [26]. Tumor tissues were examined histogically by hematoxylin and eosin (H&E) staining. And detection of apoptotic cells by terminal deoxynucleotidyl transferase dUTP nick end labeling (TUNEL) assay (Roche) was performed. All mouse experiments were approved by the animal care committee at Asan Institute for Life Science, Asan Medical Center (Seoul, Republic of Korea) and were performed in accordance with institutional guidelines and regulations.

### Statistical analysis

Datas were statistically analyzed using a two-tail Student's *t*-test, and the level of significance stated in the text was based on *P* values and we considered a *P* value lower than 0.05 as significant.

## SUPPLEMENTARY FIGURES



## Abbreviations

IAPs: Inhibitor of apoptosis proteins
MEFs: mouse embryonic fibroblasts
