# Supplementary material for: A novel small-molecule IAP antagonist, AZD5582, draws Mcl-1 down-regulation for induction of apoptosis through targeting of cIAP1 and XIAP in human pancreatic cancer
Source: Oncotarget. 2015 Aug 6;6(29):26895–908. doi: 10.18632/oncotarget.4822 (PMC4694961; doi:10.18632/oncotarget.4822)
Supplement: Supplementary file 1 [file oncotarget-06-26895-s001.pdf]

SUPPLEMENTARY FIGURES

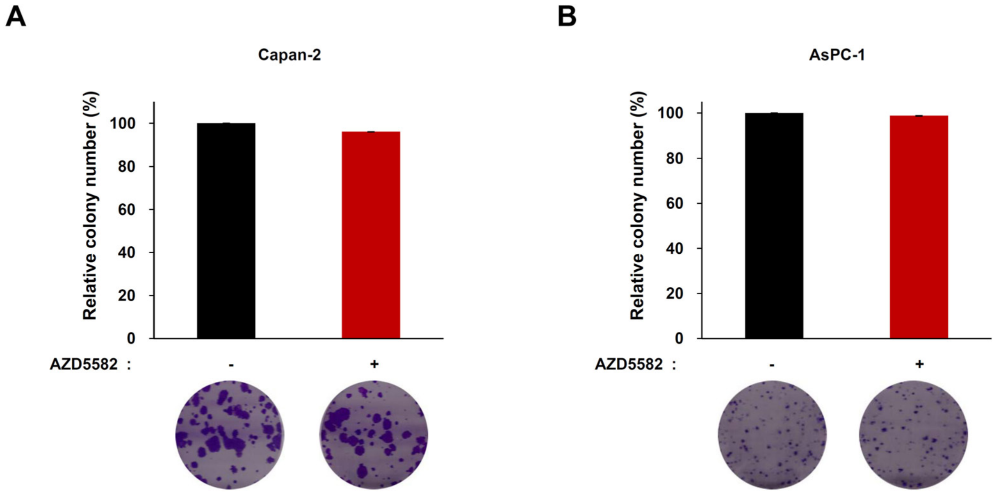

Supplementary Figure S1: Capan-2 and AsPC-1 cells were resistant to AZD5582. Colony-forming assays were performed on Capan-2 **A.** and AsPC-1 **B.** The cells were treated with 100 nM AZD5582. After 24 h, the cells were harvested, counted, and seeded into 6-well plates at a density of 3 × 10^2 cells/well. After 10–14 days the cells were fixed, stained, and photographed. The graphs present the relative number of colonies as the means ± SDs from three separate experiments performed in triplicate. *$**P < 0.01$*.

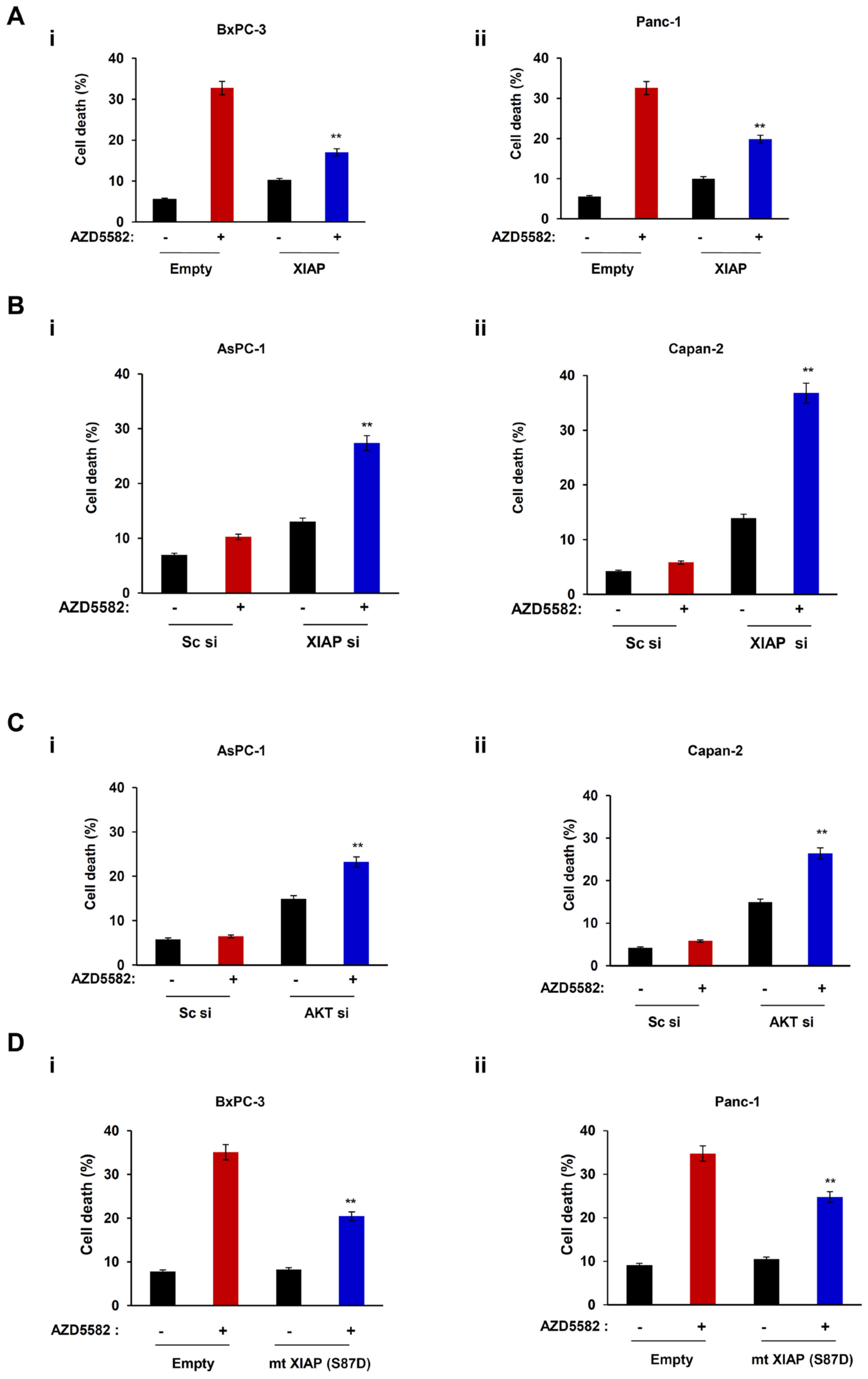

Supplementary Figure S2: Phoshporylation of XIAP by AKT mediates the resistance to AZD5582. A. BxPC-3 (left panel) and Panc-1 (right panel) were transfected with an empty vector and XIAP for 48 h then treated with 100 nM AZD5582 for 24 h. Cell death was measured by trypan blue exclusion. **$P < 0.01$. **B.** AsPC-1 (left panel) and Capan-2 (right panel) were transfected with scrambled and XIAP siRNA for 48 h then treated with 100 nM AZD5582 for 24 h. Cell death was measured using trypan blue exclusion. **$P < 0.01$. **C.** AsPC-1 (left panel) and Capan-2 (right panel) were transfected with scrambled and Akt siRNA for 48 h and treated with 100 nM AZD5582 for 24 h. Cells were harvested and cell death was measured trypan blue exclusion. **D.** BxPC-3 (left panel) and Panc-1 (right panel) were transfected with empty and mutant XIAP (S87D) for 48 h and then treated with 100 nM AZD5582 for 24 h. Cell death was measured using trypan blue exclusion. **$P < 0.01$.

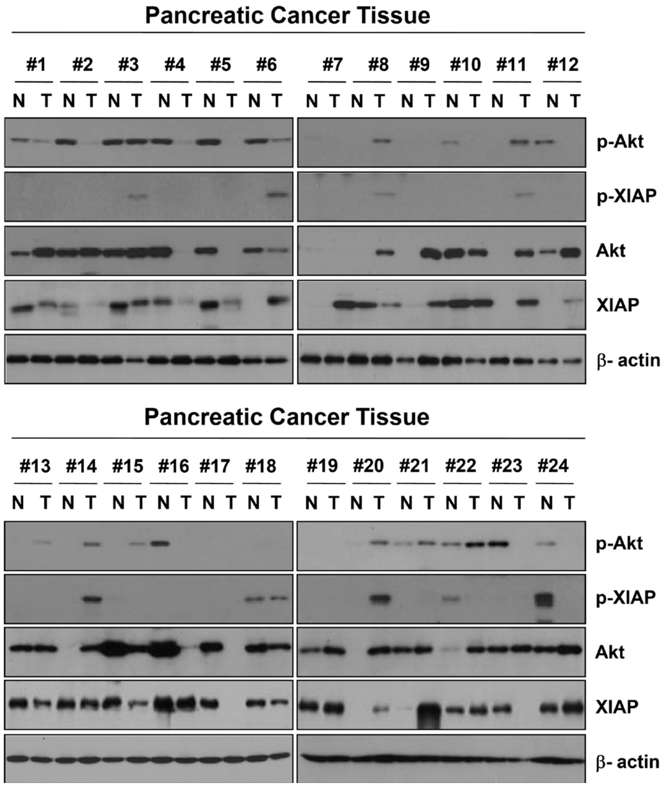

Supplementary Figure S3: pAKT and pXIAP were co-expressed in pancreatic cancer tissues. Paired normal (N) and pancreatic cancer tissues (T) were lysated and analyzed by immunoblotting against p-AKT, p-XIAP, AKT, and XIAP. β- actin was used as a loading control.

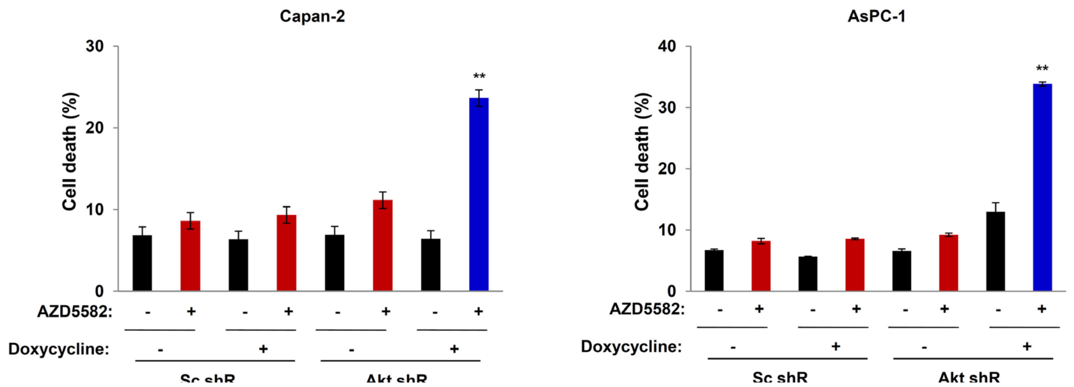

Supplementary Figure S4: Knockdown of AKT sensitizes to AZD5582 in AZD5582-resistant cells. Cells stably transfected with Dox-on inducible Akt-shRNA were treated with 1 μg/ml doxycycline in the presence/absence of AZD5582 (100 nM). Cell death was measured by trypan blue exclusion. The values are presented as the means ± SDs from three separate experiments performed in triplicate. *$**P < 0.01$*.

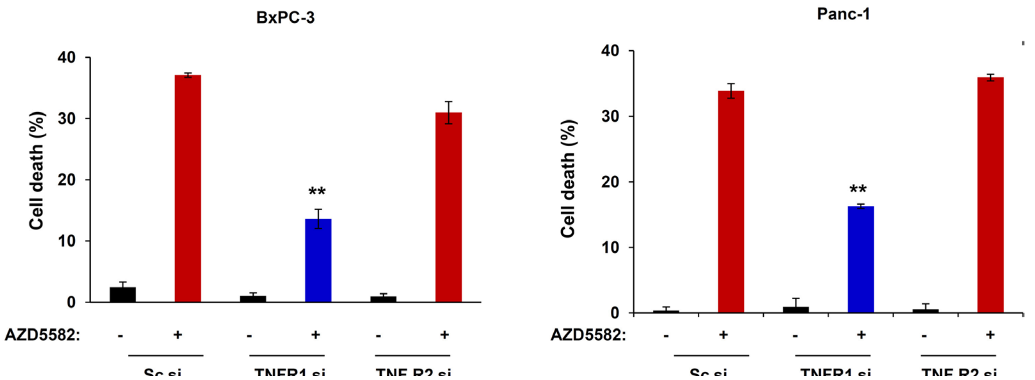

Supplementary Figure S5: TNFR1 plays important role in AZD5582-induced apoptosis. BxPC-3 and Panc-1 cells were transfected with TNFR1 or TNFR2 siRNA and then treated with 100 nM AZD5582 for 24 h. Cell death was determined by trypan blue exclusion assay. $^{**}P < 0.01$

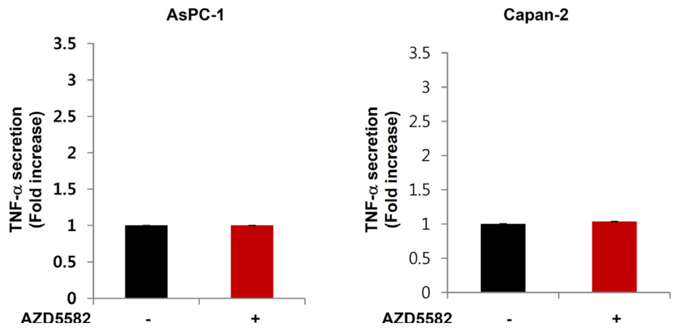

Supplementary Figure S6: AZD5582-resistant cell lines did not secret TNF-α. Capan-2 and AsPC-1 cells were treated with 100 nM AZD5582 for 24 h, and then the cell supernatants were collected and analyzed by TNF-α ELISA assays. The graph displays the means ± s.d. ***P < 0.01*.

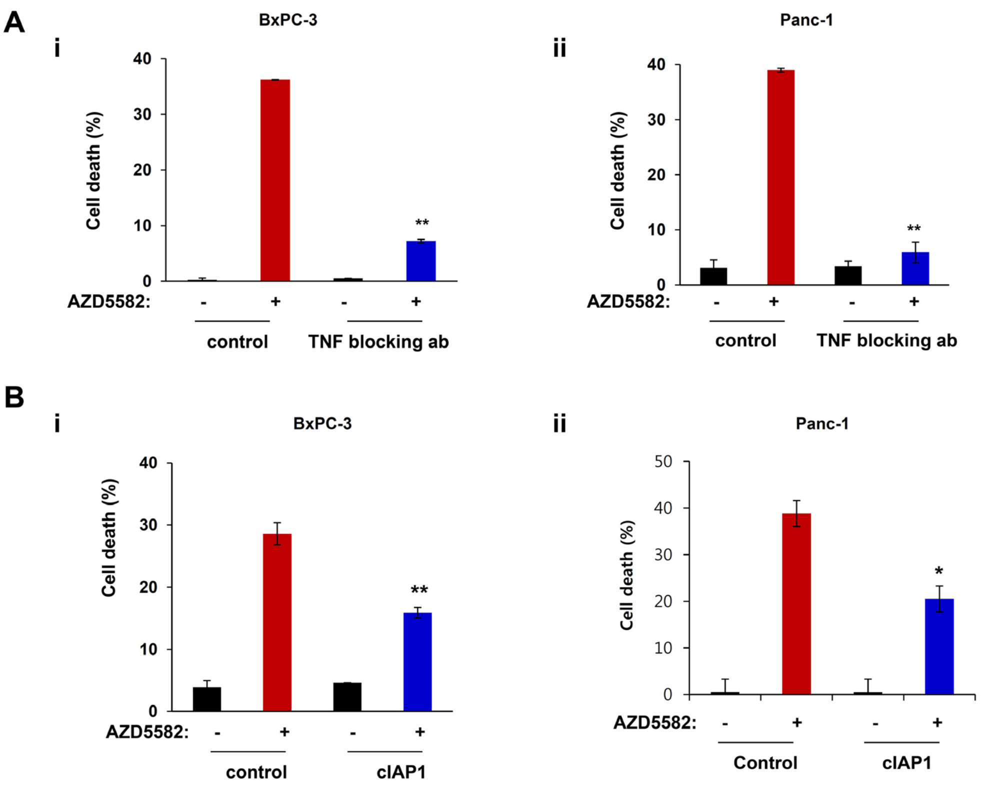

Supplementary Figure S7: AZD5582 induces TNF-α-cIAP1 dependent apoptotic pathway. A. Cells were treated with 100 nM AZD5582 and TNF-α blocking antibodies at 5 μg/ml for 24 h. Cell death was determined by trypan blue exclusion assay. *$P < 0.01$*. **B.** BxPC-3 cells were transfected with a cIAP1-expressing plasmid and then treated with 100 nM AZD5582 for 24 h. Inhibition of cell death by cIAP1 overexpression was determined using the trypan blue exclusion method. The data are the means ± s.d. **$P < 0.01$.

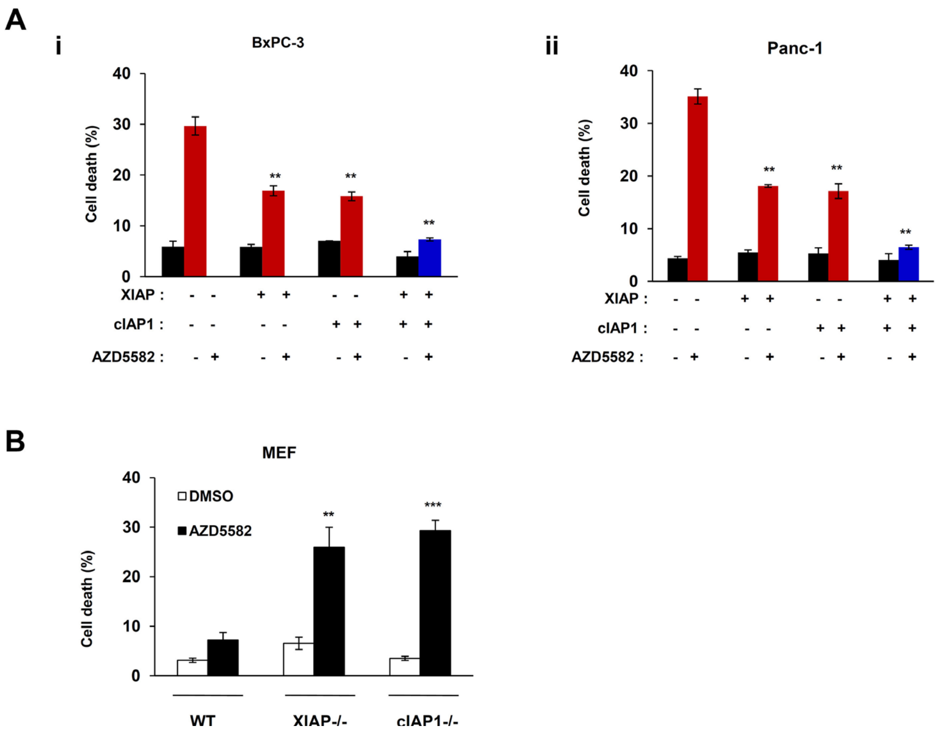

Supplementary Figure S8: Overexpression of XIAP and cIAP1 significantly inhibits AZD5582-induced apoptosis. **A.** BxPC-3 and Panc-1 cells were treated with 100 nM AZD5582 for 24 h then cells were prepared for trypan blue staining. **$P < 0.01$ **B.** Wild-type, XIAP-null and cIAP1 null MEFs were treated with optimal concentration of AZD5582 for 48 h and then cell death was determined by trypan blue exclusion assay. **$P < 0.01$. ***$P < 0.001$.

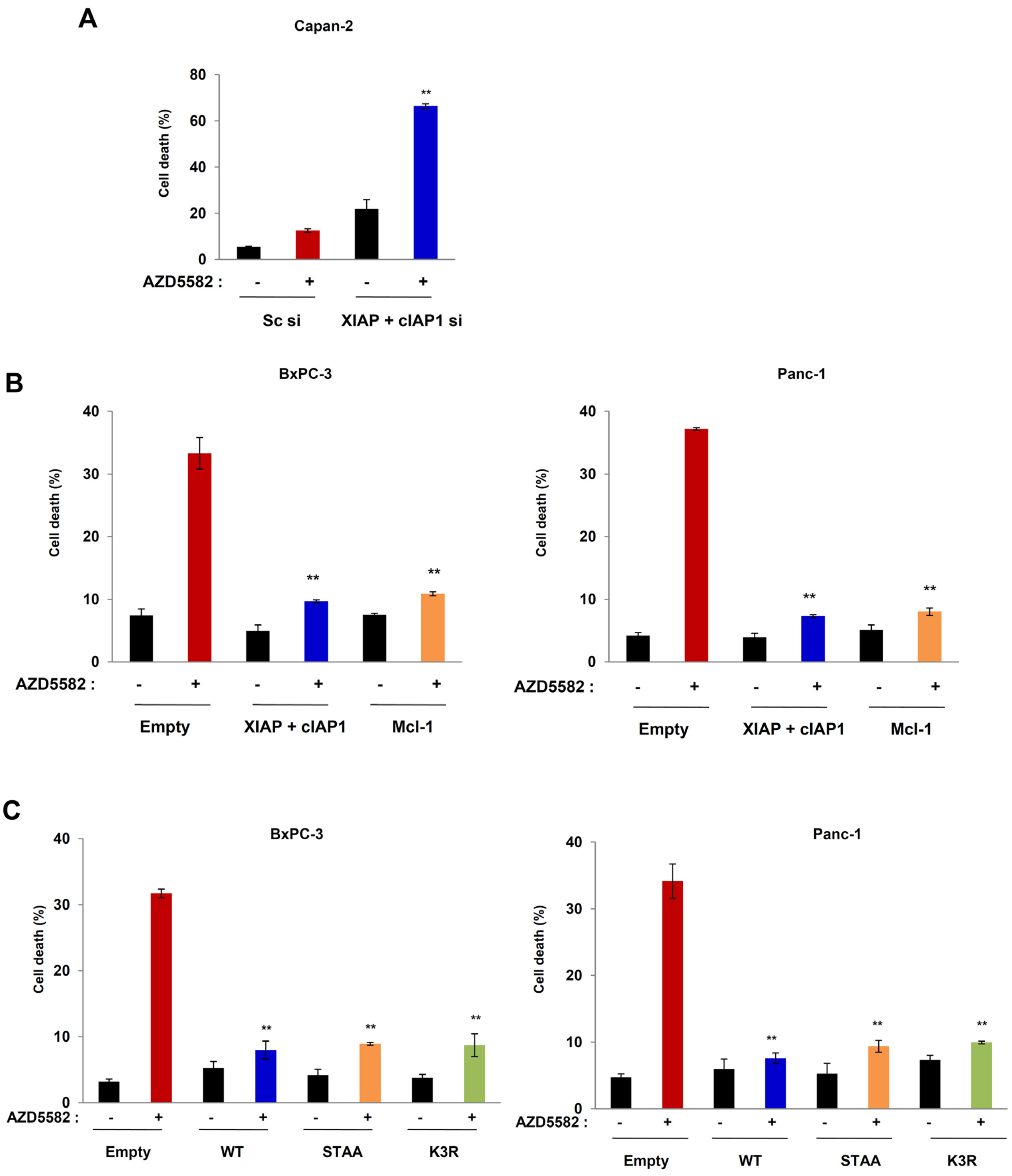

Supplementary Figure S9: Overexpression of Mcl-1 completely inhibits AZD5582-induced apoptotic cell death such as XIAP and cIAP1 expression. A. Capan-2 cells were transfected with XIAP- and cIAP1-specific siRNAs for 24 h, treated with 100 nM AZD5582 for 24 h, then harvested for trypan blue counting. $^{**}P < 0.01$. **B.** BxPC-3 and Panc-1 cells were transfected with HA-tagged XIAP and cIAP1 or myc-tagged Mcl-1 plasmid and then treated with 100 nM AZD5582 for 24 h. Cell death was determined using trypan blue exclusion assay. $^{**}P < 0.01$. **C.** BxPC-3 and Panc-1 cells were transfected with Mcl-1 wild type or STAA (Ser168 and Thr163 substituted with alanine) or K3R (K136, K194 and K197 substituted with arginine) and then treated with 100 nM AZD5582 for 24 h. The rate of cell death was quantified by trypan blue exclusion assay. $^{**}P < 0.01$.

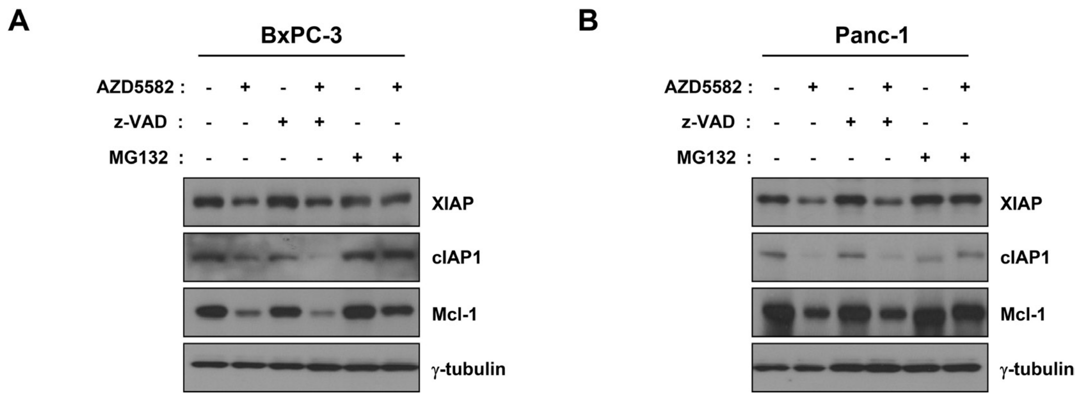

Supplementary Figure S10: AZD5582 inhibits expression of XIAP, cIAP1 and mcl-1 through a proteasome-dependent pathway but not in caspase-dependent manner. BxPC-3 **A.** and Panc-1 cells **B.** were treated with 100 nM AZD5582 for 24 h after pretreatment with 50 µM z-VAD, a pan-caspase inhibitor, or after treatment with 10 µM MG132 for 6 h. Cells were harvested and immunoblotted using anti-XIAP, anti-cIAP1, and anti-mcl-1. γ-tubulin was used as a loading control.

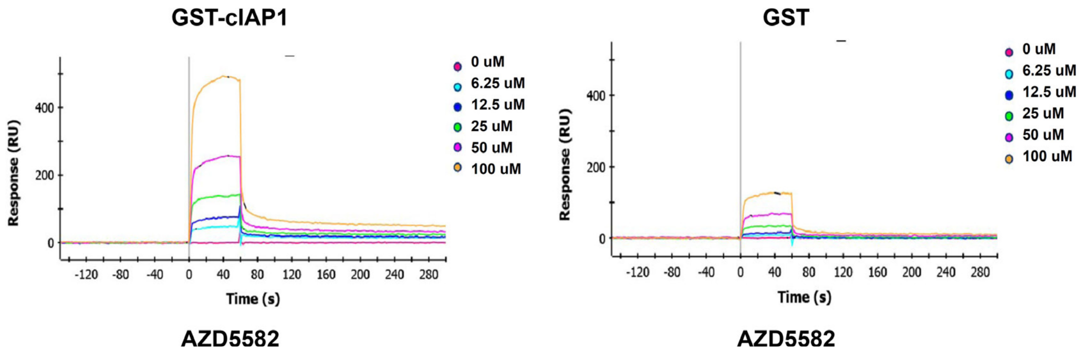

Supplementary Figure S11: AZD5582 binds to cIAP1 protein. SPR analysis confirmed that AZD5582 interacted with cIAP1 (left panel). And right panel was shown negative control.

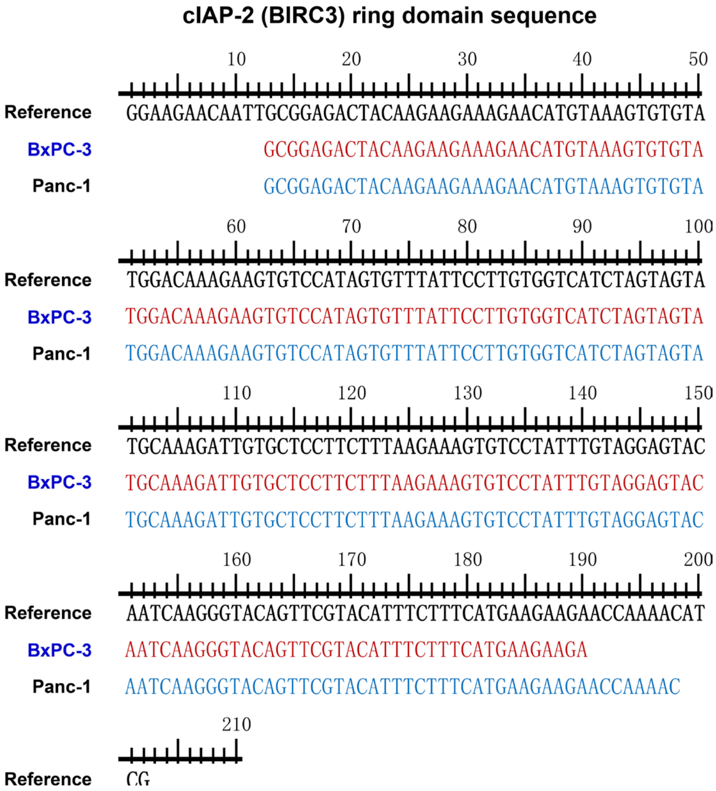

Supplementary Figure S12: The Ring domain mutation of cIAP2 in Panc-1 and BxPC-3 was analyzed by sequencing.
